# Hop Bitter Acids Increase Hippocampal Dopaminergic Activity in a Mouse Model of Social Defeat Stress

**DOI:** 10.3390/ijms21249612

**Published:** 2020-12-17

**Authors:** Yasuhisa Ano, Shiho Kitaoka, Rena Ohya, Keiji Kondo, Tomoyuki Furuyashiki

**Affiliations:** 1 Kirin Central Research Institute, Kirin Holdings Company Ltd., 1-13-5, Fukuura, Kanazawa-ku, Yokohama 236-0004, Japan; Rena_Ohya@kirin.co.jp (R.O.); kondok@kirin.co.jp (K.K.); 2 Division of Pharmacology, Kobe University Graduate School of Medicine, Kobe 650-0017, Japan; skitaoka@med.kobe-u.ac.jp (S.K.); tfuruya@med.kobe-u.ac.jp (T.F.)

**Keywords:** depression, dopamine, hippocampus, hop bitter acids, repeated social defeat stress

## Abstract

As daily lifestyle is closely associated with mental illnesses, diet-based preventive approaches are receiving attention. Supplementation with hop bitter acids such as iso-α-acids (IAA) and mature hop bitter acids (MHBA) improves mood states in healthy older adults. However, the underlying mechanism remains unknown. Since acute oral consumption with IAA increases dopamine levels in hippocampus and improves memory impairment via vagal nerve activation, here we investigated the effects of chronic administration of hop bitter acids on the dopaminergic activity associated with emotional disturbance in a mouse model of repeated social defeat stress (R-SDS). Chronic administration of IAA and MHBA significantly increased dopaminergic activity based on the dopamine metabolite to dopamine ratio in the hippocampus and medial prefrontal cortex following R-SDS. Hippocampal dopaminergic activity was inversely correlated with the level of R-SDS-induced social avoidance with or without IAA administration. Therefore, chronic treatment with hop bitter acids enhances stress resilience-related hippocampal dopaminergic activity.

## 1. Introduction

Given the increasing incidence of depression and insufficiency of depression medication, daily life diet-based preventive approaches have been gaining attention. Based on several epidemiological studies, low to moderate alcoholic beverage consumption has mental health benefits. The effects of resveratrol, a red wine ingredient, suppress depression-like behavior in socially-defeated rats and increase dopamine and serotonin levels in the prefrontal cortex (PFC) [1,2]. However, the effects of beer ingredients on emotional disturbance remain to be investigated. Social interaction is important for mental health and the activity of social interaction is closely associated with emotional disturbance and depression. An evaluation of social interaction is important to understand its effect on emotion.

According to previous studies, the hop-derived bitter compounds, iso-α-acids (IAA), increase hippocampal dopamine levels through the vagal nerves and improve scopolamine-induced memory impairment in an amnesia mouse model [3]. IAA also suppress microglial activation and brain inflammation and improve cognitive functions in an Alzheimer’s disease mouse model [4,5]. Additionally, matured hops from bitter acids (MHBA), another type of hop bitter acid, improves memory impairment in mice via the vagal nerves [6]. Based on clinical trials, MHBA supplementation improves anxiety and reduces stress marker levels in healthy adult saliva [7,8].

Vagal activation and non-steroidal anti-inflammatory drug-based treatment have therapeutic effects in patients with depression. Additionally, rodent studies have demonstrated dopamine’s role in stress-induced emotional disturbance. Dopamine exerts effects on motor, emotional, and cognitive functions through multiple brain areas including the medial PFC (mPFC), nucleus accumbens (NAc), caudate-putamen (CPu), and hippocampus. Positron emission tomography imaging studies revealed reduced dopamine transporter binding in the striatum of patients with depression [9]. In rodents, chronic environmental stress, including chronic mild stress and repeated social-defeat stress (R-SDS), alters PFC and NAc dopaminergic activity, leading to emotional disturbances [10,11,12,13,14,15,16]. Thus, we postulated that hop bitter acids might also be beneficial for emotional disturbance. Here, we aimed to investigate the effects of the hop bitter acids IAA and MHBA on dopaminergic activities associated with emotional disturbance in a mouse model of R-SDS.

## 2. Results

### 2.1. Effects of Hop Bitter Acids on Dopaminergic Activities in a Mouse Model of Social Defeat Stress

**Figure 1:**
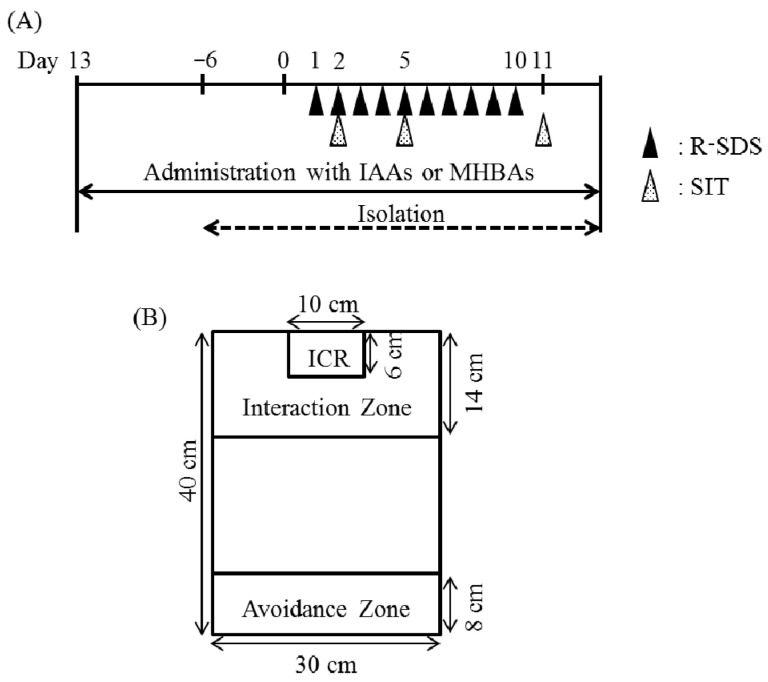
Procedures to induce repeated social-defeat stress. (**A**) The schedule of experiments in the present study. The administration of iso-α-acids (IAA) or matured hop bitter acids (MHBA) started 1 week prior to social isolation and continued throughout the experiments. After social isolation for 1 week, a male C57BL/6N mouse was subjected to repeated social-defeat stress (R-SDS) through an encounter with an aggressor Institute of Cancer Research (ICR) mouse for 10 min daily for 10 consecutive days (day 1 to day 10). On days 2, 5, and 11, each mouse was subjected to the social interaction test (SIT). The mouse was subjected to an additional R-SDS exposure on day 15 and was immediately sacrificed for the measurement of dopamine and its metabolites. (**B**) Measurement of social avoidance and social interaction in the SIT. An ICR mouse was enclosed in a metal meshwork cage (10 cm × 6 cm) at one side of the open field chamber (30 cm × 40 cm). A mouse with or without R-SDS was kept in this chamber for 2.5 min. The time spent in the avoidance zone and interaction zone was measured as the level of social avoidance and social interaction, respectively.

**Figure 2:**
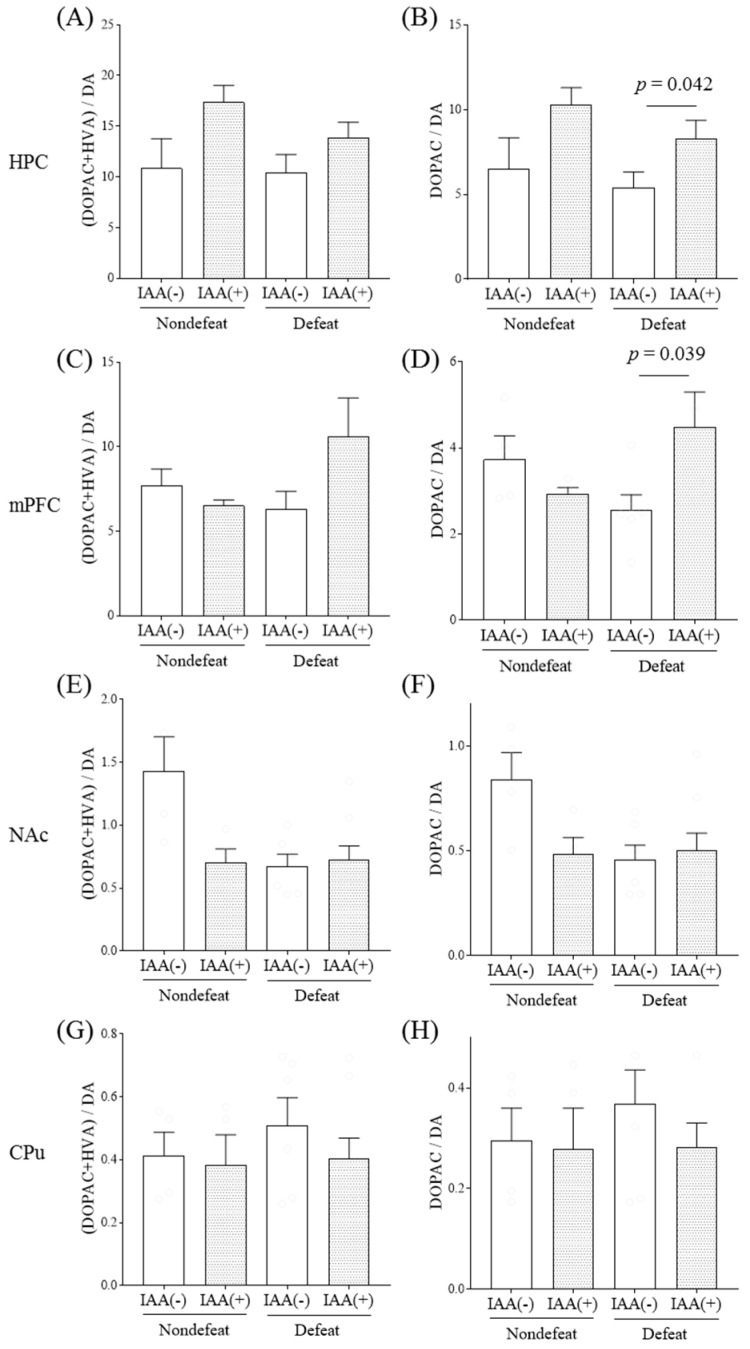
The effects of iso-α-acids on dopaminergic activities after repeated social-defeated stress. Mice were supplied with a diet containing iso-α-acids (IAA) and subjected to repeated social- defeated stress (R-SDS) and the social interaction test. The ratios of 3,4-dihydroxyphenylacetic acid (DOPAC) and homovanillic acid (HVA) to dopamine (DA) (**A**,**C**,**E**,**G**) and those of DOPAC to dopamine (DA) (**B**,**D**,**F**,**H**) in the hippocampus (HPC) (**A**,**B**), medial prefrontal cortex (mPFC) (**C**,**D**), nucleus accumbens (NAc) (**E**,**F**), and caudate-putamen (CPu) (**G**,**H**) immediately after an additional R-SDS exposure are shown. Data represent the mean ± standard error of four–eight mice per group. Significance was determined by a two-way analysis of variance with Tukey’s multiple comparisons test.

We measured the IAA effects on dopaminergic activity in several dopaminoceptive brain areas with or without R-SDS using HPLC-ECD. Since dopamine is rapidly metabolized to its metabolites, 3,4-dihydroxyphenylacetic acid (DOPAC), homovanillic acid (HVA), and 3-methoxytyramine (3-MT) upon dopamine release, the ratio of dopamine metabolites to dopamine reflects the biochemical index of dopaminergic activity. Mice were fed regular chow containing IAAs for 14 days and were subjected to R-SDS (Figure 1). In defeated mice, IAA administration increased the hippocampal ratios of (DOPAC + HVA)/dopamine (*p* < 0.05, dopamine factor and stress factor main effects and dopamine × stress interaction, two-way ANOVA; *p* = 0.040, designated comparison, Tukey-Kramer post-hoc test) and DOPAC/dopamine (*p* < 0.05, dopamine × stress interaction, two-way ANOVA; *p* = 0.042, designated comparison, Tukey-Kramer post-hoc test) (Figure 2A,B, respectively). It also increased the ratio of mPFC DOPAC/dopamine in the defeated group (*p* < 0.05, dopamine × stress interaction, two-way ANOVA; *p* = 0.039, designated comparison, Tukey-Kramer post-hoc test; Figure 2D). R-SDS alone did not significantly alter these ratios in these brain areas. Moreover, the dopamine, DOPAC, HVA and 3-MT levels were not significantly altered in these brain areas, nor did they contribute to the IAA-induced dopaminergic activity increase (Figure S1). Contrarily, in the defeated mice, neither the NAc nor CPu exhibited IAA effects on dopaminergic activity (Figure 2E–H). These results show that chronic IAA administration enhances dopaminergic activity in the hippocampus and mPFC after R-SDS.

**Figure 3:**
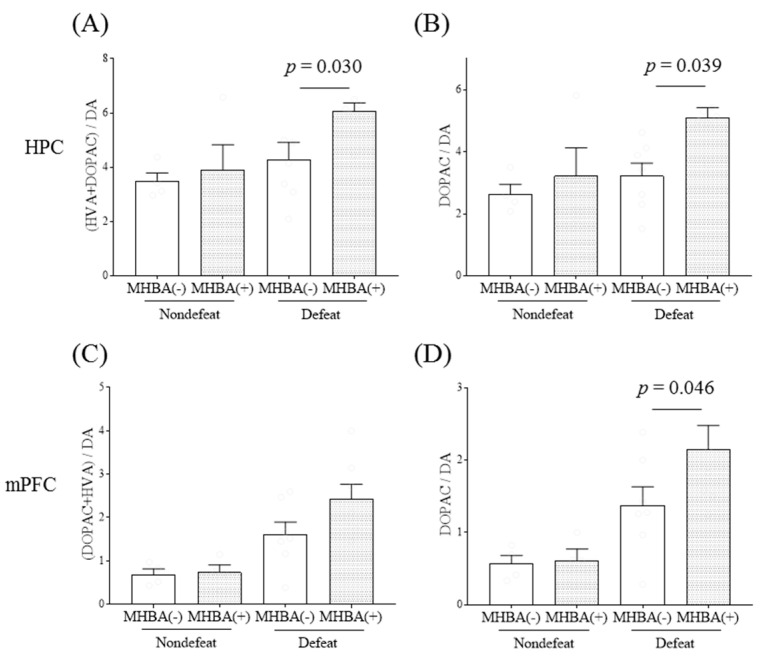
The effects of matured hop bitter acids on dopaminergic activities after repeated social-defeated stress. Mice were fed a diet containing matured hop bitter acids (MHBA) and were subjected to repeated social-defeat stress (R-SDS) and the social interaction test. The ratios of 3,4-dihydroxyphenylacetic acid (DOPAC) and homovanillic acid (HVA) to dopamine (DA) (**A**,**C**) and those of DOPAC to dopamine (DA) (**B**,**D**) in the hippocampus (HPC) (**A**,**B**) and medial prefrontal cortex (mPFC) (**C**,**D**) immediately after an additional R-SDS exposure are shown. Data represent the mean ± standard error of the mean of four–eight mice per group. Significance was determined by a two-way analysis of variance with Tukey’s multiple comparisons test.

Chronic MHBA administration similarly increased the ratios of (DOPAC + HVA)/dopamine (*p* < 0.05, dopamine × stress interaction, two-way ANOVA; *p* = 0.030, designated comparison, Tukey-Kramer post-hoc test) and DOPAC/dopamine in the hippocampus (*p* < 0.05, dopamine factor and stress factor main effects, two-way ANOVA; *p* = 0.039, designated comparison, Tukey-Kramer post-hoc test) and DOPAC/dopamine in the mPFC (*p* < 0.05, dopamine factor and stress factor main effects, two-way ANOVA; *p* = 0.046, designated comparison, Tukey-Kramer post-hoc test), with no changes in the other brain areas (Figure 3). Consistently, chronic MHBA administration significantly increased the hippocampal DOPAC levels after R-SDS (*p* < 0.05, dopamine factor and stress factor main effects, two-way ANOVA; *p* = 0.045, designated comparison, Tukey-Kramer post-hoc test; Figure S2B). Thus, both IAAs and MHBAs enhance specific dopaminergic pathways under chronic stress.

### 2.2. Effects of Hop Bitter Acids on Emotional Disturbance in a Mouse Model of Social Defeat Stress

**Figure 4:**
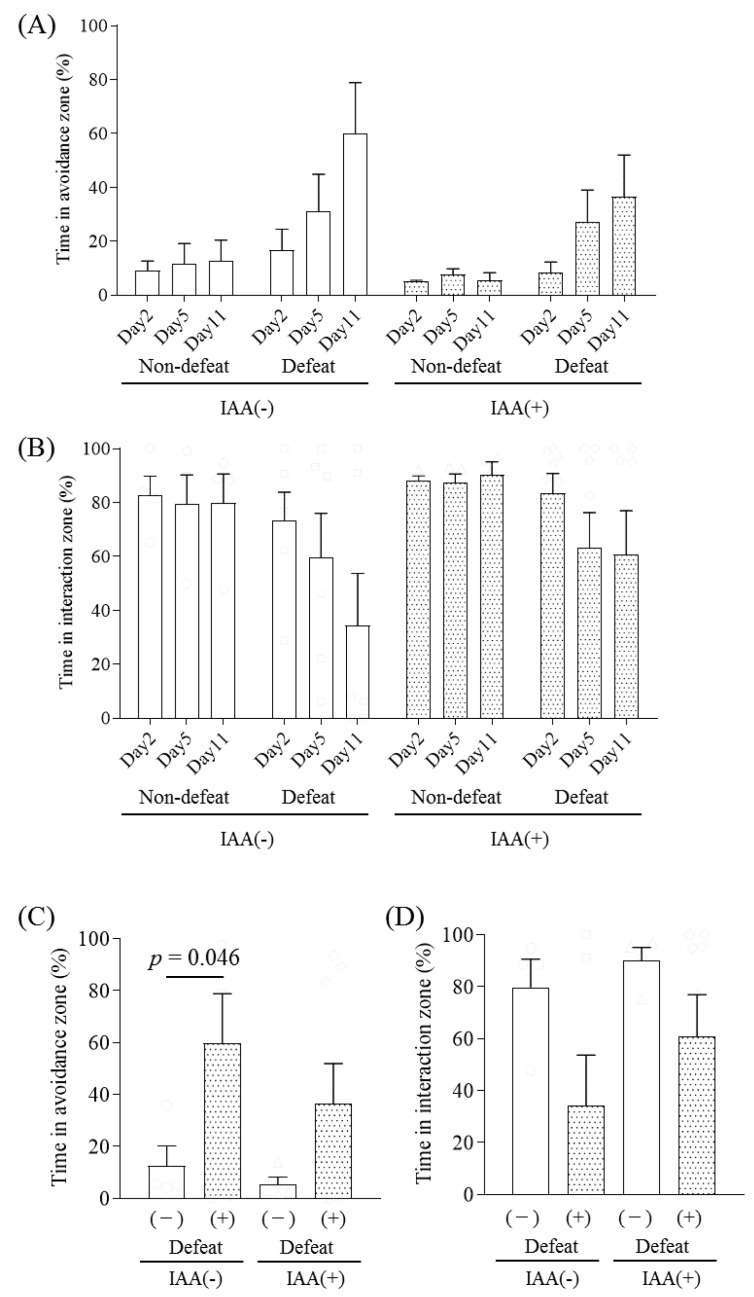
The effects of iso-α-acids on repeated social-defeated stress-induced social avoidance. C57BL/6N mice were fed a diet containing iso-α-acids (IAA) and were subjected to repeated social-defeat stress (R-SDS). The social interaction test was performed on days 2, 5, and 11. The percentage of time in the avoidance and interaction zones is shown in (**A**,**B**), respectively. Only the data points on day 11 are shown in (**C**,**D**), which were subjected to statistical analyses. Data represent the mean ± standard error of four–eight mice per group. Significance was determined by a two-way analysis of variance with Tukey’s multiple comparisons test.

Since dopamine regulates stress-induced emotional disturbance, as based on previous studies [11,17,18,19], we also examined the effects of IAAs on R-SDS-induced social avoidance. R-SDS increased the time in the avoidance zone and decreased the time in the interaction zone in the SIT without or with chronic IAA treatment, even though chronic IAA treatment appeared to reduce the proportion of defeated mice (Figure 4C,D).

**Figure 5:**
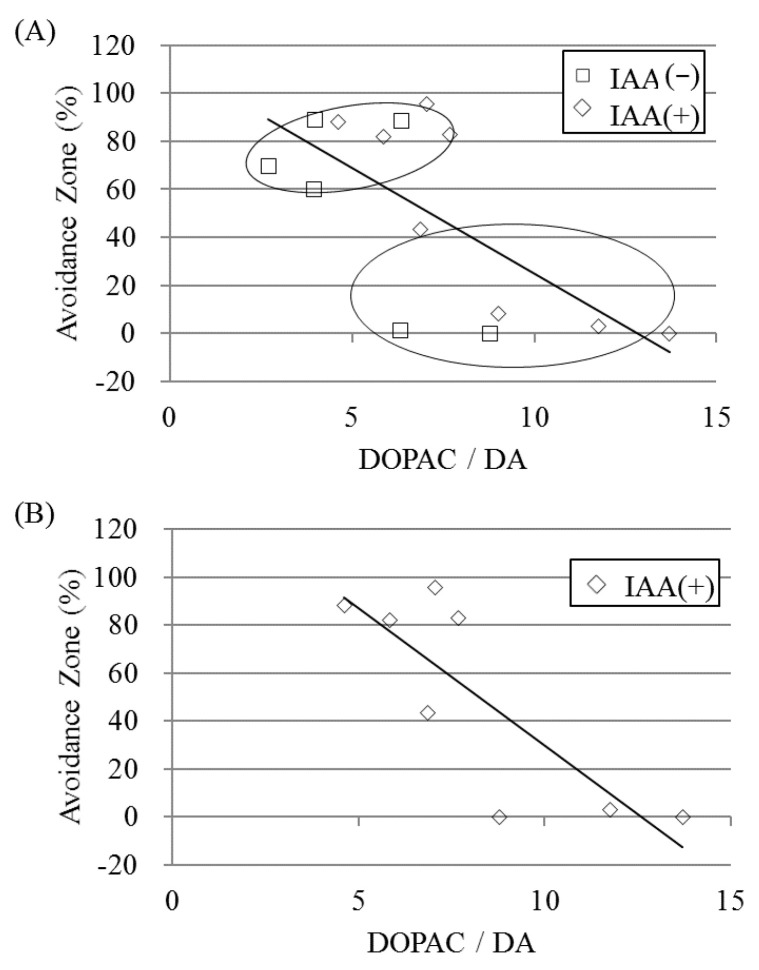
Negative correlations of hippocampal dopaminergic activity and social avoidance after repeated social-defeat stress. The relationships between the percentage of time in the avoidance zone during the social interaction test and the ratio of 3,4-dihydroxyphenylacetic acid (DOPAC) to dopamine (DA) are shown. The data points of the defeated mice with and without chronic IAA treatment are shown in (**A**). Only those with chronic IAA treatment are shown in (**B**). Spearman’s rank correlations were analyzed in the respective plots.

To determine the behavioral relevance of the IAA-induced enhancement of dopaminergic activity, we analyzed the correlation between the ratio of dopamine metabolites to dopamine and social avoidance. With and without chronic IAA treatment, the ratio of hippocampal DOPAC/dopamine was inversely correlated with social avoidance (Spearman’s rank correlation; *r* = −0.674, *p* = 0.008, Figure 5A and *r* = −0.812, *p* = 0.014, Figure 5B). Thus, IAAs enhance stress resilience-based hippocampal dopaminergic activity.

## 3. Discussion

We previously reported that IAA increase mouse hippocampal dopamine content and release and improve memory impairment [3]. However, the IAA effects on emotional disturbance-related dopaminergic activity remain to be investigated. Here, we have demonstrated for the first time that hop bitter acids increase hippocampal dopaminergic activity associated with stress resilience in an R-SDS mouse model.

IAA and MHBA, the bitter beer components, activate the intestine’s bitter taste receptors and activate the vagal nerve [20]. IAA and MHBA activate the vagal nerve without absorption. IAA-induced vagal nerve activation stimulates mouse hippocampal dopamine content and release and improves scopolamine-induced memory impairment [3]. The effects are attenuated by vagotomy, systemic dopamine D1 receptor antagonist-based treatment, and hippocampal dopamine D1 receptor knockdown. Thus, it has been suggested that hop bitter acids, including IAA and MHBA, increase dopamine synthesis and activity via the brain-gut axis resulting in cognitive improvement [6]. Here, based on the dopamine metabolite to dopamine ratios, chronic IAA treatment increased hippocampus and mPFC dopaminergic activity after R-SDS via vagal nerve activation. Chronic IAA treatment via chow feeding did not increase hippocampal dopamine levels similar to acute IAA treatment via intragastric feeding. Thus, whether the brain-gut axis via the vagal nerve is involved in dopaminergic enhancement via chronic and acute IAA treatment warrants testing. Nonetheless, our study demonstrates that chronic and acute treatments with hop bitter acids enhance dopaminergic activity in the hippocampus, especially under chronic stress.

Based on previous studies, R-SDS induces dopamine-associated dysregulation, involving reduced dopaminergic activity in the hippocampus, which is associated with the development of mood-related behavioral changes [21,22]. Accordingly, we found that dopaminergic activity in the hippocampus inversely correlates with R-SDS-induced social avoidance with or without chronic IAA treatment. It has been reported that the dopamine D1 receptor in mature granule cells in the hippocampus is crucial for the antidepressant-like effects of fluorexetine and concomitant hippocampal neurogenesis [23]. Hippocampal neurogenesis is crucial for the behavioral effects of chronic antidepressant treatment [24]. Thus, similarly, chronic IAA treatment could increase hippocampal neurogenesis by enhancing dopaminergic activity. Despite this similarity between IAA- and antidepressant-induced dopaminergic enhancement, unlike antidepressants, chronic IAA treatment did not significantly ameliorate R-SDS-induced social avoidance. However, neither the role of hippocampal dopamine nor that of the hippocampus has been implicated in R-SDS-induced social avoidance. Chronic stress, including R-SDS, induces hippocampus-dependent behavioral changes, such as increased anxiety-like behavior (elevated plus maze test), novelty-suppressed feeding, and impaired object memory (novel object recognition test). Whether the IAA-induced behavioral effects influence these other behavioral measures remains to be investigated.

Clinical studies have reported that MHBA supplementation improves the healthy adult mood state. The MHBA group exhibited statistically significant lower anxiety scores in the Profile of Mood States-2 than the placebo group in a randomized, placebo-controlled trial [7]. Here, dopaminergic activities in the hippocampus and mPFC were enhanced by chronic MHBA and IAA treatment. Indeed, IAA and MHBA have a common β-carbonyl moiety [25] and compounds with a β-carbonyl moiety improve memory impairment via vagal activation [3,6] and prevent inflammation in the brain [26]. Thus, we propose that hippocampal dopaminergic activity enhancement is a common property of hop bitter acids, which may clinically contribute to the beneficial effects of chronic supplementation with hop bitter acids on emotional disturbances. In the current study, mice were fed an AIN-93M diet with 0 or 0.05% (*w*/*w*) IAA or MHBA. Each mouse (average body weight is 25 g) intake approximately 4 g of AIN-93M, which contains 2 mg IAA or MHBA (80 mg/kg). According to the calculation of supplementation from mouse to human, a human weighing 60 kg needs to intake 31.8 mg of IAA or MHBA per day. We previously demonstrated that supplementation with 35 mg MHBA improved mood state in a clinical trial [7]. The results in the current study supports to elucidate the mechanisms of the previous clinical trial.

The current study has some limitations. We did not evaluate the involvement of dopamine in R-SDS model mice, so further studies need to evaluate the effects of IAA and MHBA on R-SDS model mice using a dopamine receptor antagonist. In the current study, we measured DA, DOPAC, 3-MT, and HVA as dopamine metabolites, but did not measure the other metabolites such as DOPAL. Since there are no reports on the involvement of these metabolites on emotional disturbance in R-SDS model mice, we did not measure other metabolites, but the recent study report DOPAL as a neurotoxin [27]. To elucidate the effects of IAA and MHBA on dopamine metabolism further, we need to measure more metabolites in the brain regions of R-SDS model mice.

Since hop bitter acids are easy and safe to consume daily via beverages, including nonalcoholic beer, the dopaminergic enhancement by these agents might be a practical approach for preventing mood and anxiety disorders. Further clinical studies for the people with high stress or anxiety will help to develop a solution to improve their mood state.

## 4. Materials and Methods

### 4.1. Preparation of IAA

a-Acids consist predominantly of 3 congeners: cohumulone, humulone, and adhumulone. During the brewing process, they are each isomerized into two epimeric isomers: cis- and trans-IAAs. A purchased isomerized hop extract stocked in potassium carbonate at 828 mM (Hopsteiner, Mainburg, Germany) with 30.5% (*w*/*v*) IAA comprising trans-isocohumulone (1.74% *w*/*v*), cisisocohumulone (7.61% *w*/*v*), trans-isohumulone (3.05% *w*/*v*), cis-isohumulone (14.0% *w*/*v*), trans-isoadhumulone (0.737% *w*/*v*), and cis-isoadhumulone (3.37% *w*/*v*) was used to isolate individual IAAs by high-performance liquid chromatography (HPLC), as previously described [4].

### 4.2. Preparation of MHBA

MHBA, including the major components 40-hydroxyallohumulinones, 40-hydroxyalloisohumulones, tricyclooxyisohumulones-A, hulupones, and humulinones, were prepared from hop pellets [25,28].

### 4.3. Animals

Seven-week-old male C57BL/6N and Institute of Cancer Research (ICR) mice (retired from breeding) were purchased from Japan SLC and maintained at Kobe University. On arrival, groups of four mice were housed in a specific pathogen-free, temperature- and humidity-controlled vivarium under a 12-h light/12-h dark cycle (light, 6:00 a.m. to 6:00 p.m.) with free access to chow and water. All animal care and use procedures complied with the National Institutes of Health Guide for the Care and Use of Laboratory Animals and were approved by Kobe University Graduate School of Medicine’s Animal Care and Use Committees (1 December 2017, Approval ID: P150304-R5). All the experiments were prepared and performed from December 2017 to January 2018. Mice were euthanized by placing them in a CO_2_ chamber (Taiyo Nippon Sanso, Tokyo, Japan). All efforts were made to minimize suffering.

### 4.4. Repeated SDS (R-SDS)

Mice were subjected to R-SDS as previously described [17]. Before R-SDS, ICR male mice were screened for their aggressiveness against a different C57BL/6N mouse for 3 min daily for 3 days. We evaluated aggression by measuring latency and the number of attacks during this period. We used mice that exhibited stable aggression. The R-SDS schedule is described in Figure 1A. Before administering R-SDS, male C57BL/6N mice were individually housed with free access to food and water for 1 week. Mice were fed a standard rodent diet, American Institute of Nutrition (AIN)-93G or AIN-93G, containing 0.05% (*w*/*w*) IAAs or MHBAs for 14 days before R-SDS. These mice were then transferred to the home cage of a male ICR mouse for 10 min daily for 10 days. The pairs of defeated and aggressor mice were randomized daily to minimize the variability in aggressiveness of the aggressor mice. After the 10-min defeat episode, the mice were returned to their home cages and isolated until they were subjected to R-SDS the next day. Control mice were transferred to a new cage and allowed to freely explore for 10 min. All data were included in the analyses.

### 4.5. Social Interaction Test (SIT)

Following R-SDS, the defeated and control mice were tested using the social interaction test (SIT) [17,29]. Briefly, they were placed in an open field chamber where a new male ICR mouse was enclosed in a metal meshwork at one end (Figure 1B). The mice were allowed to freely explore for 150 s during the recording. They were habituated to the same chamber in the absence of ICR mice for 150 s before the SIT. The chamber was divided into an interaction zone (closest to the ICR mouse), a middle zone, and an avoidance zone (farthest from the ICR mouse). The time each mouse spent in each zone was analyzed using the SMART video tracking software (PanLab, Harvard Apparatus).

### 4.6. Monoamine Analysis

To evaluate the levels of dopamine and its metabolites in the respective brain regions after R-SDS, the brains were collected immediately after the last R-SDS exposure. They were rapidly removed from decapitated mice and chilled in ice-cold Dulbecco phosphate-buffered saline. They were sliced at 0.5 mm intervals using a mouse brain slicer matrix. Punches (diameter, 1.5 mm) were used to sample tissues from the mPFC, NAc, and CPu. The hippocampus was cut away from a coronal slice of the brain at the bregma level (−3.3 to −3.8 mm) with a surgical knife. The collected tissues were quickly frozen in liquid nitrogen and stored at −80 °C until assay. During the assay, the tissue sample was homogenized in 0.2 M perchloric acid (Wako) containing 100 μM ethylenediaminetetraacetic acid (EDTA)-2Na (Sigma-Aldrich) [30]. The samples were then centrifuged, and the supernatant was analyzed by HPLC using the Eicompak SC-5ODS and PrePak columns (Eicom, Kyoto, Japan) with an electrochemical detection (ECD) unit. The mobile phase consisted of 83% 0.1 M acetic acid in citric acid buffer (pH 3.5), 17% methanol (Wako), 190 mg/mL sodium 1-octanesulfonate sodium (Wako), and 5 mg/mL EDTA-2Na. Regarding the ECD, the applied voltage was 750 mV vs. an Ag/AgCl reference electrode.

### 4.7. Statistical Analysis

The data represent the mean, and the error bars indicate the standard error of the mean (SEM). Data were analyzed using a two-way analysis of variance (ANOVA) followed by Tukey’s multiple comparisons test. Correlation was analyzed using Spearman’s rank correlation. All statistical analyses were performed using GraphPad Prism 7 (GraphPad Software, Inc., CA, USA). *p* < 0.05 was considered statistically significant.

## Supplementary Materials

Supplementary materials can be found at https://www.mdpi.com/1422-0067/21/24/9612/s1. Figure S1: The effects of iso-α-acids on dopamine and its metabolites after repeated social-defeated stress. Mice were supplied with a diet containing iso-α-acids (IAAs) and subjected to repeated social-defeated stress (R-SDS) and social interaction tests. The levels of dopamine (DA) (A,E,I,M), 3,4-dihydroxyphenylacetic acid (DOPAC) (B,F,J,N), homovanillic acid (HVA) (C,G,K,O), and 3-methoxytyramine (3-MT) (D,H,L,P) in the hippocampus (HPC) (A–D), medial prefrontal cortex (mPFC) (E–H), nucleus accumbens (NAc) (I–L), and caudate-putamen (CPu) (M–P) immediately after an additional R-SDS exposure. (Q) Chromatogram of HPLC in a representative sample from CPu of mice received R-SDS. Data represent the mean ± standard error of four–eight mice per group. Significance was determined by a two-way analysis of variance with Tukey’s multiple comparisons test. Figure S2: The effects of matured hop bitter acids on dopamine and its metabolites after repeated social-defeated stress. Mice were fed a diet containing matured hop bitter acids (MHBA) and were subjected to repeated social-defeat stress (R-SDS) and social interaction tests. The levels of dopamine (DA) (A,E), 3,4-dihydroxyphenylacetic acid (DOPAC) (B,F), homovanillic acid (HVA) (C,G), and 3-methoxytyramine (3-MT) (D,H) in the hippocampus (HPC) (A–D) and medial prefrontal cortex (mPFC) (E–H) immediately after an additional R-SDS exposure are shown. Data represent the mean ± standard error of four–eight mice per group. Significance was determined by a two-way analysis of variance with Tukey’s multiple comparisons test.

## Abbreviations

CPu	Candate-putamen
DA	Dopamine
DOPAC	3,4-dihydroxyphenylacetic acid
HVA	Homovanillic acid
IAA	Iso-α-acids
MHBA	Mature hop bitter acids
NAc	Nucleus accumbens
PFC	Prefrontal cortex
R-SDS	Repeated social defeat stress
