# Supplementary figures and images for: Hop Bitter Acids Increase Hippocampal Dopaminergic Activity in a Mouse Model of Social Defeat Stress
Source: Int J Mol Sci. 2020 Dec 17;21(24):9612. doi: 10.3390/ijms21249612 (PMC7766517; doi:10.3390/ijms21249612)

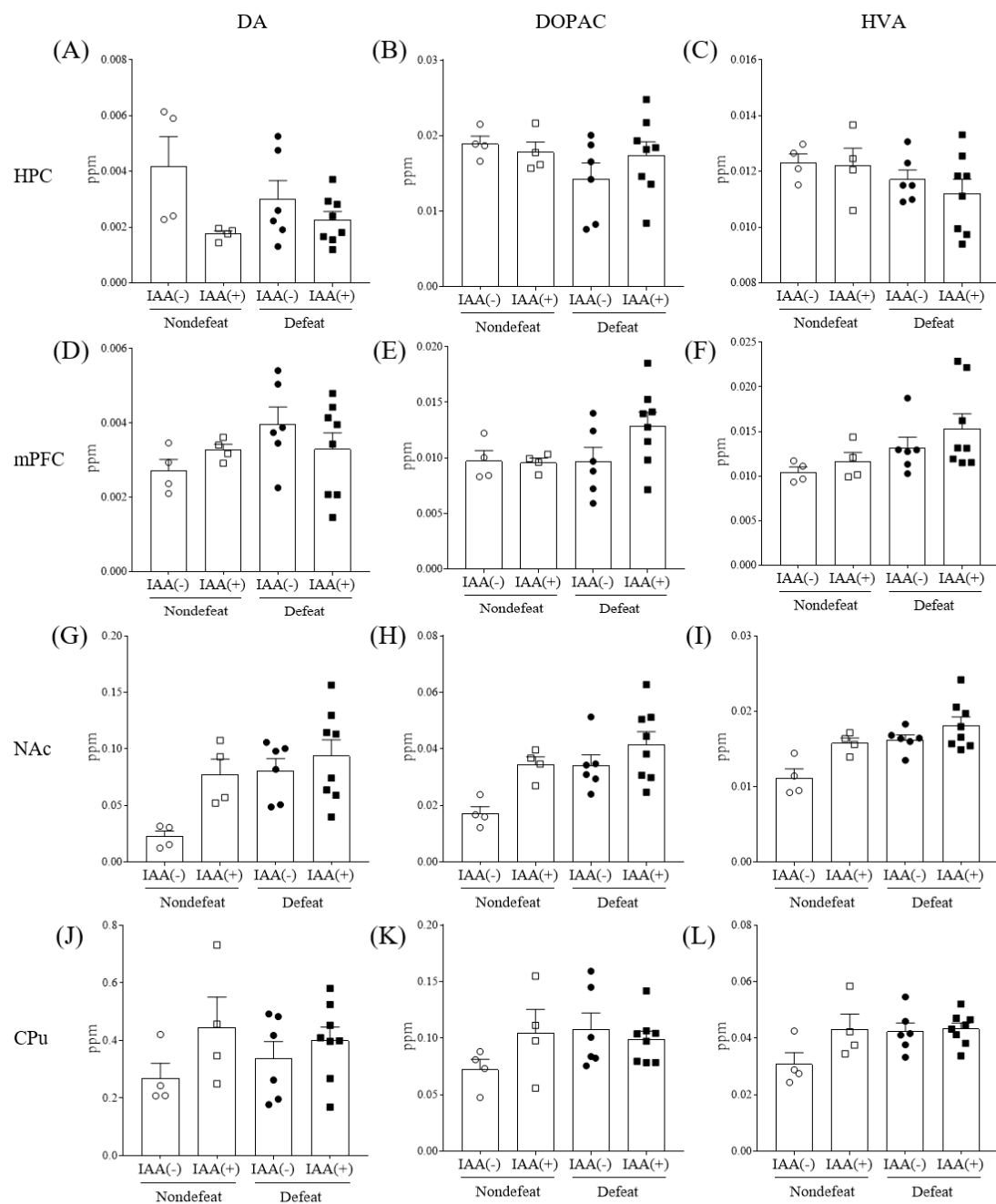

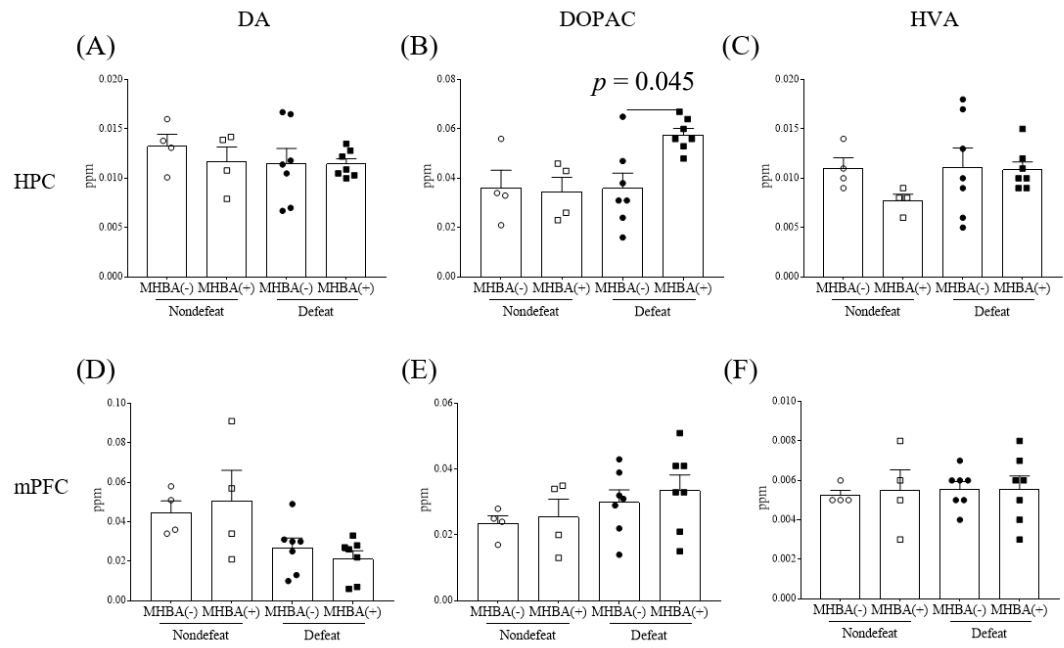

Supplement: Supplementary file 1 [file ijms-21-09612-s001.pdf]
